# Molecular classification of breast cancer: A retrospective cohort study

**DOI:** 10.1016/j.amsu.2019.11.021

**Published:** 2019-12-06

**Authors:** Fatma Khinaifis Al-thoubaity

**Affiliations:** Department of Surgery, Faculty of Medicine, King Abdulaziz University, Jeddah, Saudi Arabia

**Keywords:** Breast carcinoma, Molecular subtypes, Histological grade, Axillary lymph node metastases

## Abstract

**Objectives:**

The study was aimed to determine the distribution of various breast cancer molecular subtypes in Saudi Arabia. Further, association between these subtypes and different epidemiological features was assessed.

**Methods:**

A retrospective study was conducted between January 2012 and December 2018, at the King Abdul Aziz University Hospital. A total of 740 cases of breast cancer, using immunohistochemistry, were classified into 4 major molecular subtypes: luminal A, luminal B, HER2-positive, and triple negative. Chi-squared test was performed to evaluate the relationship between these subtypes and clinico-pathological features.

**Results:**

Luminal A (58.5%) subtype was the most prevalent, followed by triple negative (16%), luminal B (14%), and HER2-positive (11.5%). The average age of the patient at the time of diagnosis was found to be 49 years with an average tumor size of 3.2 cm. Out of all cases, 85% of cases were ductal, while 11.4% were lobular. 66.6% showed axillary lymph node metastases. While, 77% of lobular carcinomas were found almost exclusively in the luminal A and triple negative tumor subtype, 69.5% had modified radical mastectomy.

**Conclusions:**

Luminal A tumor was the most prevalent subtype, while HER2-positive was the least prevalent. Luminal A tumors were mostly associated with lobular carcinomas. HER2-positive and triple negative tumors showed higher histological grade and larger tumor size at the time of diagnosis. These tumors were commonly found in women below the age of 50 years. Carcinoma-in-situ was less prevalent in HER2-positive tumors. Furthermore, a strong association was observed between axillary lymph node status and molecular subtypes.

## Highlights

• The prognosis to treatment of breast cancer is influenced by a number of aspects.
• Molecular subtypes of breast cancer are strong prognostic and predictive factors.
• Luminal A tumor was found as the most common molecular subtype among patients.
• A strong association between lymph node status and molecular subtypes was observed.

## 1 Introduction

Breast cancer is a heterogeneous disorder representative of numerous subcategories of several cellular compositions, molecular alterations as well as clinical behavior. A number of factors such as histological grade, type and size of tumor, lymph node metastasis, estrogen receptor (ER), progesterone receptor (PR) and human epidermal growth factor receptor 2 (HER2/neu), influence the prognosis and response to the treatment of cancer. One of the most commonly observed malignancies around the world is the Breast cancer (BC) [1]. In the United States (US), an estimate of about a quarter of million new cases of BC were recorded in the year 2014, which in turn accounted for about 14% of all the new cancer cases [2]. Around 50,285 new cases of BC were diagnosed in the United Kingdom, accounting for nearly 15% of all new cancer cases [3]. Incidence of BC is much lower in the Kingdom of Saudi Arabia (KSA) than the Western world. However, in the Saudi women, breast cancer is the most common malignancy and accounts for about a quarter of the newly diagnosed cancer in females [4]. Approximately 27.4% of the newly diagnosed female cancers (5,378) in Saudi were breast cancer patients (ranked first) as reported by the National Cancer Registry, KSA in the year 2010. It is worthy to note that the incidences of breast cancer is lower in KSA (age standardized rate per 100,000 is 29.6) as compared to the worldwide average (age standardized rate per 100,000 is 43.1), but it nonetheless represents a significant fraction of the cancer related fatality in women [1]. In US, median diagnosis age for breast cancer is 61 [5], whereas, in Arab countries breast cancer is diagnosed at a younger age. In KSA, mean age for the diagnosis of breast cancer is 49 years [4,6], and this cancer is generally found to be aggressive and locally advanced [7]. Despite the fact that breast cancer mortality has moderately reduced due to currently available treatments, it is estimated that more than 450,000 deaths occur annually due to breast cancer worldwide [4,6]. Molecular subtypes of breast cancer based on histological grade and lymph node metastases, are strong prognostic and predictive factors. Consequently, classifying breast cancer into relevant molecular subtypes is an important aspect of therapeutic decision-making. Classical immunohistochemistry (IHC) markers such as ER, PR and HER2 play a crucial role in molecular subtyping [8]. Newer methods like gene expression profiling using complementary DNA microarrays have been developed, which are therapeutically important for molecular classification. Immunohistochemical analyses of tumors on the basis of status of ER, PR, and HER2 is used in clinical practice, and this method is easier, cost-effective and provide similar results for molecular subtypes [9,10]. Immunohistochemistry based molecular subtyping of tumors is now considered as the main stay to predict susceptibility of tumor to hormonal therapy and subsequent Trastuzumab therapy [9,11]. Newer classification methods are also being developed that are based on immunohistochemical, genetic and molecular findings [11,12]. Availability of hormone (estrogen and progesterone) receptor markers marked the beginning of molecular classification about 30 years ago. HER2/neu based determination techniques then followed the earlier developments. Further, a new study mandated the molecular classification of human BC by initially dividing BC into four major classes: luminal-like, basal-like, normal-like, and HER-2 positive [13]. Subsequently, luminal class was divided into luminal A and luminal B classes, thereby resulting in addition of a fifth class of BC [14]. According to the St. Gallen Consensus 2011, molecular subtypes of breast cancer can be classified into Luminal A (ER+/PR+/HER2-/lowKi-67); Luminal B (ER+/PR+/HER2-/+/high Ki-67); HER2-overexpression (ER-/PR-/HER2+) and triple negative breast cancers/TNBCs (ER-/PR-/HER2-) [9]. Basal-like subtype of breast cancer referred to as TNBC was found to be positive for basal marker (CK5/6) expression [15,16]. Thus, it is important that use of techniques enabling molecular subtyping in clinical practice would provide more accurate information about patient-specific prognosis, risk of relapse and probability for pathological complete response. One of the major advantages would be the ease of identification of patients for whom the benefits of neoadjuvant therapy outweigh the risks, i.e. it will result in improved risk stratification. Furthermore, aggressive treatment strategy or increased surveillance can be designed for the patients who face an increased risk of relapse. In the present study, we aimed to analyze the prevalence of breast cancer subtypes in the western region of KSA and associated clinico-pathological features, which will eventually increase our understanding of breast cancer and lead to effective healthcare management.

## 2 Materials and methods

### 2.1 Data collection

**Fig. 1:**
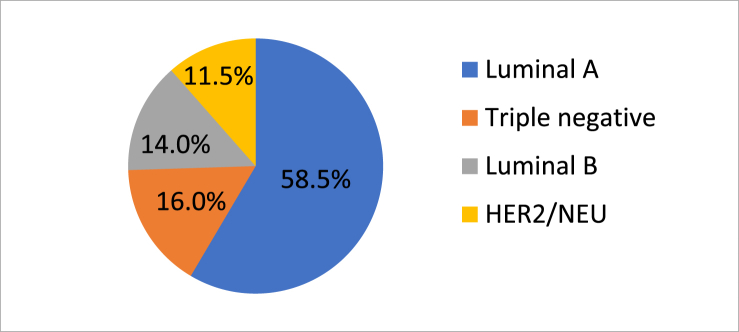
Molecular subtype of breast carcinoma in KAUH (2012–2018).

This is a retrospective analysis based on data retrieved from the Pathology Department at the King Abdul Aziz University Hospital across a seven-year study period. All the histopathology reports of patients diagnosed with primary invasive breast cancer from January 2012 to December 2018 were utilized. This analysis was conducted with prior approval from the Institutional Review Board at the King Abdul Aziz University Hospital. The study has been reported in line with the STROCSS criteria [17]. Histological grade was assessed according to the Nottingham modification of the Bloom-Richardson system. The criteria for inclusion of patients into this analysis were as follows: (a) patients with invasive breast carcinoma, (b) patients with available histological grade and lymph node status, and (c) available formalin-fixed, paraffin embedded samples with good quality. Male patients and recurrence cases were excluded from the analysis leading to a total of 740 finally approved cases. We obtained the following parameters for each patient: age at the time of diagnosis, tumor size, histopathological subtype, Scarff-Bloom-Richardson (SBR) grade, presence or absence of carcinoma in-situ component, lymph node status, immunohistochemical profile of the hormonal receptors ER and PR, and immunohistochemical profile of HER2 in the invasive malignant cells. The tumor size measurement was retrieved from ultrasound reports of the breast prior to the biopsy or using reports from other radiological modalities. After size assessment, tumors were grouped into three categories: ≤2 cm, >2 but ≤5 cm, and >5 cm. Tumor grade evaluation was carried out based on the established Elston-Ellis modification of the SBR system, which relies on histochemical features such as the percentage of tubular differentiation, the presence of nuclear atypia/pleomorphism and the number of mitoses [18]. The status of the lymph node metastasis was determined either using radiological modalities or from the evaluation of axillary lymph nodes obtained at mastectomy. Thereafter, the number of lymph nodes was determined along with the number of lymph nodes positive for metastasis. The ER, PR and HER2 tests were scored according to the Guidelines of the College of American Pathologists [18]. Positive ER or PR is considered when ≥1% of invasive malignant cells that exhibit nuclear staining or immunoreactivity. Additionally, for ER and PR, another semi-quantitative scoring system called the Allred (Quick) scoring system was employed to ascertain the proportion of stained cells and assess the intensity of the nuclear staining [18]. The HER2 test was scored from 0 to 3 + in which: score 0 or 1 are negative; 2 + is equivocal; and 3 + is positive. A 3 + score is given when an intense full circumferential cytoplasmic membrane staining is observed in more than 10% of invasive malignant cells. Specimens showing equivocal HER2 staining were sent for further examination with the help of fluorescent in situ hybridization (FISH) and their results were documented. We classified the breast cancer Fig. 1 into four molecular subtypes according to ER, PR, and HER2/neu status: 1) luminal A (ER and/or PR positive and HER2/neu negative), 2) luminal B (ER and/or PR positive and HER2/neu positive), 3) HER2-positive (ER and PR negative and HER2/neu positive), and 4) triple negative (ER, PR, and HER2/neu negative) [9,10].

### 2.2 Statistical analysis

The Statistical Package for Social Sciences software version 21.0 (IBMCorp., Armonk, NY, USA) was used for statistical analyses. Descriptive statistics, frequency, and percentages of categorical variables have been reported. We examined the association between the molecular subtypes and age at diagnosis, tumor size, histopathological subtype, grade, presence of foci of in situ carcinoma, and nodal status using Chi-squared test for categorical variables. We computed the odds ratio (OR) where appropriate and constructed the 95% confidence interval (CI). The results were considered statistically significant if the p-value was <0.05.

### 2.3 Ethical considerations

There were minimal ethical implications and issues since it is a retrospective study. Patient identity and confidentiality were protected by assigning each patient a specific serial number. Moreover, no one except the investigating research team accessed the patients’ records. We obtained prior approval from the Institutional Review Board since a consent form was not applicable to our study. The study is registered with Research Registry; research registry5151.

## 3 Results

In the present study, a total of 740 breast cancer cases were examined, and the average age of the patient at time of diagnosis was 49 years (standard deviation of 12.28). Most cases (n = 629, 85%) were ductal, a few of them (n = 84, 11.4%) were lobular, while the rest of the cases were of other histological types that included medullary, tubular, mucinous, metaplastic, adenoid cystic, and encysted papillary carcinoma. Most of the cases of cancer that were detected were mildly differentiated (n = 274, 37.1%) followed by moderately differentiated (n = 248, 33.5%). The average size of the tumor at the time of diagnosis was found to be 3.2 cm (standard deviation of 1.92). Most patients presented a tumor size between 2 and 5 cm (n = 362, 48.9%), while some of the patients (n = 315, 42.6%) exhibited a tumor size <2 cm (Table 1). At diagnosis, most breast cancer tumors exceeded 2 cm in maximum dimension. The patients whose age was less than 50 years had higher probability of displaying greater tumor size (p = 0.036, OR = 1.613, 95% CI, 1.030–2.526) than the patients who were in their sixties or older. More than half of the cases (n = 493, 66.6%) that were investigated showed lymph node metastases (Table 1). Positive ER immunostaining was found in 70.8% of the cases and the PR in 63.8%. HER2 immunostaining was found positive in 18.7% of the cases and equivocal in 22.8%. FISH testing was performed for the equivocal cases, and it was established that 34.2% of the equivocal cases were HER2 positive. Luminal A was the most prevalent subtype (n = 434, 58.5%) followed by, triple negative (n = 117, 16%), luminal B (n = 104, 14%), and HER2-positive (n = 85, 11.5%)Fig. 1. Table 1, Table 2 showed the distribution of various clinical and pathological characteristics among different molecular subtypes. Higher frequency (66–70.5%) of HER2-positive and triple negative tumors was observed as compared to luminal tumors, in the patients whose age was less than 50 years. But, these results were statistically insignificant (p = 0.124). HER2-positive (n1) samples showed tumor mass size greater than 2 cm in 82.5% of the cases. On the other hand, triple negative tumors (n2) had a tumor mass size greater than 2 cm in 75% patients (p = 0.018), where the majority of the tumor sizes ranged between 2 and 5 cm (n1 = 26, 65% and n2 = 31, 59.6%), while the rest showed tumor size of more than 5 cm (n1 = 7, 17.5% and n2 = 8, 15.4%) (p = 0.057). Additionally, these subtypes showed aggressive microscopic features and approximately two-thirds of these subtypes demonstrated poorly differentiated carcinomas. Furthermore, HER2-positive tumors were observed to be displayed least frequently as an in situ component (41.2%, p = 0.026). On the other hand, lobular carcinomas were found almost exclusively in the luminal A and triple negative tumor subtype (77%, p = 0.002). Around 69.5% had modified radical mastectomy and 30.5% had breast conserving therapy. Survival rate was found to be 90%. Patients with recurrence of breast cancer were excluded from study.

**Table 1:** The distribution of clinico-pathological characteristics, according to the hormonal and molecular subtypes in 740 women with invasive breast cancer.

Characteristics'	Luminal A	Luminal B	HER-2 positive	Triple negative	Total
Total	434 (58.5%)	104 (14%)	85 (11.5%)	117 (16%)	740(100%)
*Age (years)*					
≤50	236 (54.3%)	61 (58.7%)	50 (58.8%)	90 (76.9%)	437(59%)
>50	198 (45.7%)	43 (41.3%)	35 (41.2%)	27(23.1%)	303(41%)
*Tumor size (cm)*					
≤2	221(50.9%)	32(30.8%)	30(35.3%)	32 (27.4%)	315(42.6%)
>2 – ≤ 5	200 (46.1%)	52 (50%)	40(47.1%)	70 (59.8%)	362(48.9%)
>5	13 (3%)	20(19.2%)	15 (17.6%)	15(12.8%)	63(8.5%)
*Lymph nodes metastasis*					
Negative	192(44.2%)	20(19.2%)	15(17.7%)	20(17.1%)	247(33.4%)
*Positive*					
*1–3*	52(12%)	50(48.1%)	50(58.8%)	50(42.7%)	202(27.3%)
*>4*	190(43.8%)	34(2.7%)	20(23.5%)	47(40.2%)	291(39.3%)

**Table 2:** The distribution of histopathological characteristics, based on hormonal & molecular subtypes in 740 women with invasive breast cancer.

Characteristics'	Luminal A	Luminal B	HER-2 positive	Triple negative	Total
Total	434 (58.5%)	104(14%)	85 (11.5%)	117 (16%)	740(100)
*Histology*					
Ductal	343 (78.6%)	88 (84.6%)	84 (98.8%)	114 (97.4%)	629 (85%)
Lobular	73 (16.7%)	10 (9.6%)	1 (1.2%)	0	84 (11.4%)
Others	18 (4.7%)	6 (5.8%)	0	3 (2.6%)	27 (3.6%)
*Tumor grade*					
Grade 1	270 (62.2%)	4 (3.8%)	0	0	274 (37.1%)
Grade II	116 (26.7%)	60 (57.7%)	35 (41.2%)	37 (31.6%)	248 (33.5%)
Grade III	48 (11.1%)	40 (38.5%)	50 (58.8%)	80 (68.4%)	218 (29.4%)
*Carcinoma* in situ					
Percent	334 (77%)	54 (51.9%)	35 (41.2%	90 (76.9%)	227 (30.7%)
*Absent*	100 (23%)	50 (48.1%)	50 (58.8%)	27 (23.1%)	513 (69.3%)

## 4 Discussion

In this study, we investigated the distribution of various molecular subtypes of breast cancer from patients at King Abdul Aziz University Hospital, and also evaluated the differences in clinico-pathological features between these subtypes. Our study found that the average age of the patients was 49 years, which was in accordance with national average as reported by the Saudi Arabian Cancer Incidence Report [4,19]. Most of our cases (54.3%) were detected in women who were younger than 50 years of age, which is again similar to a recently reported study from Oman [20]. These results were in contrast to the observations in US where 65.1% of the reported cases were found in women older than 55 years of age, as evident from the Surveillance, Epidemiology, and End Results (SEER) Cancer Statistics Review [5]. This difference in age group and earlier onset may be a consequence of a lack of adequate healthcare systems in the Middle East compared to the US. It is also important to note that in the present study 42.6% of the patients had a tumor size <2 cm, while in countries like the US and Poland it is 58.4% and 51.9%, respectively [2,21]. This implies that there is aggressive presentation and delayed diagnosis in the Saudi population, which may be due to lack of awareness in the community about breast cancer as well as absence of a comprehensive screening program. The findings in our study relating to the distribution of molecular subtypes was found to be in concurrence with the results obtained from studies originating from various Asian and Western countries (Table 3). In most of the studies pertaining to the distribution of the breast cancer, luminal A was found to be the most prevalent subtype (Table 3). Even so, any minor geographical variations of tumor subtypes proportions could be related to environmental factors, genetic factors and/or technological disparity. In contrast to our study, about half the cases (52.8%) in the study by Tamimi et al. [22] were found to be triple negative, while luminal tumors represented 28.5%. Furthermore, in our study we observed that occurrence of lobular carcinomas was majorly found in the luminal A and triple negative group (77%, p = 0.002), which in a way matched the findings of Tamimi et al. [22] and Yang et al. [21]. However, according to the data presented in the Egyptian [15] and Norwegian studies [23], only 55% of lobular carcinomas were luminal A. Moreover, poorly differentiated carcinomas, the HER2-positive and triple negative tumors were observed in greater frequency [14,24]. In comparison with the luminal A subtype, these subtypes are found to be associated with an increased frequency of a larger tumor size [25,26], and with a young age group [25,27]. In the current study, we identified a strong association between the different molecular subtypes and lymph node status, with 82.3% positive lymph node involvement in HER2-positive cases. Although, there were multiple studies that failed to detect such an association [25,28], there were other studies that identified a high degree of association between lymph node metastasis with HER2-positive tumors and lower frequency with basal-like tumors [18,29]. This contradiction may be due to the fact that there are studies that indicate the tumor subtype may be intrinsic and therefore only loosely associated with lymph node status. In contrast to 434 patients with luminal A tumors (77%) and 117 patients with triple negative tumors (76.9%), only 41.2% of HER2-positive tumors (p = 0.026) displayed an in-situ component. In another study, 45 cases of the luminal tumors (n = 124) showed an in-situ component [30]. The role of mammography in the detection of the various molecular subtypes has also been suggested in a recent study [31]. 69.5% had modified radical mastectomy and 30.5% had breast conserving therapy; a higher percentage of patients had mastectomy because of advanced cancer. In our study, the survival rate was 90%. Despite providing many interesting observations, our study has certain limitations. One of the limitation are due to the unavailability of Ki67 [32], a cellular marker that differentiates between non-HER2 expressing luminal B from luminal A tumors [9]. Similarly, limitations are in the detection of Basal-like tumors, a subset of triple negative tumors, due to the absence of cytokeratin 5/6 [29]. Moreover, there is a discrepancy rate of 39% in the molecular classification of tumors by immunohistochemistry and gene expression [33].

**Table 3:** The distribution of molecular subtypes of breast carcinomas by immunohistochemistry in various regional & western countries.

Variables	Mehdi et al.	Yang et al.	Cheng et al.	Vallejos et al.	Fourati et al.	Carey et al. African American	Carey et al. Non-African American	Riyadh	Jeddah
Setting	Oman	Poland	China	Peru	Tunisia	California, USA		Saudi Arabia	
Number of patients	452	804	628	1198	966	196	300	357	740

Years	2006–2010	2000–2003	2007–2010	2000–2002	2007–2009	1993–1996	1993–1996	2010–2014	2012–2018

Luminal A	34.7%	69.0%	46.5%	49.3%	50.7%	47.4%	54.0%	58.5%	58.5%
Luminal B	15.9%	6.0%	17.0%	13.%	13.4%	12.7%	17.3%	14.5%	14%
HER2/NEU	24.1%	8.0%	15.0%	16.2%	13.4%	8.2%	5.6%	12.3%	11.5%
Triple negative	25.3%	18.0%	21.5%	21.3%	22.5%	31.6%	23.0%	14.8%	16%

## 5 Conclusion

Our study has revealed that the most common tumor subtype are the luminal A tumors, followed by triple negative tumors. Luminal A and triple negative tumors were found to be closely linked with increased frequency of lobular carcinomas. The HER2-positive and triple negative tumors were associated with an increased frequency of large tumor size and poorly differentiated carcinomas as well as more aggressive manifestation of cancer. Additionally, HER2-positive tumors were less frequently observed in carcinoma, in situ. We also observed a strong correlation between lymph node status and molecular subtypes. This phenomenon needs to be examined, urgently addressed, and early screening mammography should be established in KSA. We also recommend in-depth investigation into the risks factors associated with different molecular subtypes of breast carcinoma in KSA. Further, it is also important to investigate the effect of different breast cancer subtypes on the prognosis and survival of the patient.

## Provenance and peer review

Not commissioned, externally peer reviewed.

## Ethical approval

This retrospective review approved by Department of Surgery and Faculty of Medicine.

NO 6/D/39/10851.

For Dr fatma Al thoubaity to review files of patient's with breast cancer.

## Sources of funding

We would like to express our gratitude to the working staff at the King Fahad Research Centre for providing help with data collection.We would also like to thank Professor Adnan Merdad since most of the data used in this study were from his patients.

This work was not supported or funded by any agency.

## Author contribution

It is a single author paper and the author performed all the tasks.

## Trial registry number

1. Name of the registry: Research Registry.

2. Unique Identifying number or registration ID: researchregistry5151.

3. Hyperlink to the registration (must be publicly accessible): https://www.researchregistry.com/browse-the-registry#home/

## Guarantor

Author: Fatma Khinaifis Al-thoubaity.

## Consent

No consent obtained as consent form was not applicable to our study.

This is retrospective study, collecting data from file of patients.

## Declaration of competing interest

Author has no conflict of interest. This is retrospective study data collected from files of patient from King Fahad research Centre in King Abdul-Aziz University Hospital, Jeddah, Saudi Arabia.
